# Annual acknowledgement of manuscript reviewers

**DOI:** 10.1186/s12934-015-0215-7

**Published:** 2015-03-15

**Authors:** Antonio Villaverde

**Affiliations:** Institut de Biotecnologia i de Biomedicina, Universitat Autònoma de Barcelona Bellaterra, Barcelona, 08193 Spain; Department de Genètica i de Microbiologia, Universitat Autònoma de Barcelona, Bellaterra, Barcelona, 08193 Spain; CIBER de Bioingeniería, Biomateriales y Nanomedicina (CIBER-BBN), Bellaterra, Barcelona, 08193 Spain

## Abstract

The Editors of *Microbial Cell Factories* would like to thank all our reviewers who have contributed to the journal in Volume 13 (2014).

**Usama Ramadan Abdelmohsen**

Germany

**Pratul Agarwal**

United States of America

**Tatiana Aguiar**

Portugal

**Beth Ahner**

United States of America

**Rinji Akada**

Japan

**Andres R. Alcantara**

Spain

**Pietro Alifano**

Italy

**Josef Altenbuchner**

Germany

**Ricardo Amils**

Spain

**Largus Angenent**

United States of America

**Jozef Anne**

Belgium

**Josephine Anthony**

India

**Anjali Apte-Deshpande**

India

**Joaquin Arino**

Spain

**Herb Arst**

United Kingdom

**Ranjana Arya**

India

**Magnus Ask**

Austria

**Shota Atsumi**

United States of America

**Bernhard Auer**

Austria

**Loredana Baccigalupi**

Italy

**Herwig Bachmann**

Netherlands

**Linquan Bai**

China

**Fengwu Bai**

China

**Antonio O Ballasteros**

Spain

**Uttam Banerjee**

India

**François Baneyx**

United States of America

**Imrich Barak**

Slovakia

**Paolo Barghini**

Italy

**Rodolphe Barrangou**

United States of America

**Stefan Barth**

Germany

**Gerold Barth**

Germany

**Karl Bayer**

Austria

**Carmela Belloch**

Spain

**Heather Benson**

Australia

**Gabriele Berg**

Austria

**Maria Celia Bertolini**

Brazil

**Maurizio Bettiga**

Sweden

**Jean-michel Betton**

France

**Sanjoy K. Bhattacharya**

United States of America

**Rebekka Biedendieck**

Germany

**Roslyn Bill**

United Kingdom

**Virendra Bisaria**

United States of America

**Lars Mathias Blank**

Germany

**Matthew Blankschien**

United States of America

**Mark Blenner**

United States of America

**Eckhard Boles**

Germany

**Irina Borodina**

Denmark

**Nicole Borth**

Austria

**Luca Brambilla**

Italy

**Filipe Branco dos Santos**

Netherlands

**Paola Branduardi**

Italy

**Christelle Breton**

France

**Stephanie Bringer-Meyer**

Germany

**Peter Bron**

Netherlands

**Claudia Büchel**

Germany

**Jochen Büchs**

Germany

**Andreas Burkovski**

Germany

**Tania Caetano**

Portugal

**Pinar Calik**

Turkey

**Carole Camarasa**

France

**Andrea Camattari**

Austria

**Shawn Campagna**

United States of America

**Flavia Canellas**

Switzerland

**Olivia Cano**

Spain

**James Carothers**

United States of America

**Margarida Casal**

Portugal

**Eda Celik**

Turkey

**Hyung Joon Cha**

Korea, South

**Anju Chadha**

India

**Mustapha Chamekh**

Belgium

**Verawat Champreda**

Thailand

**Tom Chappell**

United States of America

**Thomas Chasteen**

United States of America

**Jean-Marc Chatel**

France

**Guo-Qiang Chen**

China

**Rachel Chen**

United States of America

**Jong Hyun Choi**

Korea, South

**Parkson Chong**

United States of America

**Hubert Cieslinski**

Poland

**Victor Cifuentes**

Chile

**Donatella Cimini**

Italy

**Evaldas Ciplys**

Lithuania

**Tony Collins**

Portugal

**Jonas Contiero**

Brazil

**Jose Luis Corchero**

Spain

**Pierre Cornelis**

Belgium

**Donald Court**

United States of America

**Don Cowan**

South Africa

**Marc Creus**

Switzerland

**Gilles Curien**

France

**Rosa M. Cusido**

Spain

**Simon Cutting**

United Kingdom

**Jean-Marc Daran**

Netherlands

**Tina Daviter**

United Kingdom

**Carla de Carvalho**

Portugal

**Jan-Willem de Gier**

Sweden

**Stefan de Kok**

Netherlands

**Ario de Marco**

Slovenia

**Truus de Vrije**

Netherlands

**Frank Delvigne**

Belgium

**Arnold Demain**

United States of America

**Yu Deng**

United States of America

**Emma Denham**

United Kingdom

**Diana Di Gioia**

Italy

**Jean-Paul di Rago**

France

**Ulrich Dobrindt**

Germany

**Pedro Domingues**

Portugal

**Marina Donova**

Russian Federation

**Vera Doroshenko**

Russian Federation

**Martin Dragosits**

Austria

**Whasley Duarte**

Brazil

**Mara Duncan**

United States of America

**Gary Dunny**

United States of America

**Ravi Durvasula**

United States of America

**Lothar Eggeling**

Germany

**Sven-Olof Enfors**

Sweden

**Adelfo Escalante**

Mexico

**Eduardo Espeso**

Spain

**Thaddeus Ezeji**

United States of America

**Victor Fedorenko**

Ukraine

**Xueyang Feng**

United States of America

**Francisco Jesus Fernandez Morales**

Spain

**David Fewer**

Finland

**Daniela Fiocco**

Italy

**Doreen Fischer**

Germany

**Alastair Fleming**

Ireland

**Francisco Florencio**

Spain

**Debora Foguel**

Brazil

**Stephen Fong**

United States of America

**Luis Fonseca**

Portugal

**Laetitia Fontaine**

Belgium

**Pierre Fontanille**

France

**Bob Ford**

United Kingdom

**Jan Förster**

Germany

**Jochen Förster**

Denmark

**Marco Fraaije**

Netherlands

**Giovanna Franciosa**

Italy

**Kristi Frank**

United States of America

**Carl Johan Franzén**

Sweden

**Annie Frelet-barrand**

France

**Karl Friehs**

Germany

**Xiaozhi Fu**

China

**Hendrik Fuchs**

Germany

**Toshiaki Fukui**

Japan

**Fernando G. Fermoso**

Spain

**Claudia Galinha**

Portugal

**Giuseppe Gallo**

Italy

**Hector Garcia Martin**

United States of America

**Elena Garcia-Fruitos**

Spain

**Rozenn Gardan**

France

**Carlos Garrido**

Spain

**Brigitte Gasser**

Austria

**Anli Geng**

Singapore

**George Georgiou**

United States of America

**N. Louise Glass**

United States of America

**Florian Glauche**

Germany

**Benjamin Glick**

United States of America

**Monika Glinkowska**

Poland

**Francesc Godia**

Spain

**Yong Jun Goh**

United States of America

**Katsuya Gomi**

Japan

**Luciana Goncalves**

Brazil

**M. Isabel González-Siso**

Spain

**Johann Gorgens**

South Africa

**Guillermo Gosset**

Mexico

**Minakshi Grover**

India

**Amy Grunden**

United States of America

**Markus Grütter**

Switzerland

**Jose Guillamón**

Spain

**Chandrasekhar Gurramkonda**

United States of America

**Patrick Hallenbeck**

Canada

**Dietmar Haltrich**

Austria

**Colin Harwood**

United Kingdom

**Caroline Harwood**

United States of America

**Martin Haslbeck**

Germany

**Tomohisa Hasunuma**

Japan

**Feras Hatahet**

United States of America

**Annele Hatakka**

Finland

**Michael Hecker**

Germany

**Kristina Hedfalk**

Sweden

**Elmar Heinzle**

Germany

**Peter Henderson**

United Kingdom

**Colin Hill**

Ireland

**Krzysztof Hinc**

Poland

**Bjoern Hock**

Germany

**Helge Holo**

Norway

**Pascal Hols**

Belgium

**Sylvester Holt**

Belgium

**Yiguo Hong**

China

**Bo Hu**

United States of America

**Yolina Hubenova**

Bulgaria

**William J. (Jim) Hunter**

United States of America

**Tuomas Huovinen**

Finland

**Michael Hust**

Germany

**Hiroyuki Imanaka**

Japan

**Adam Iwanicki**

Poland

**Laura Jarboe**

United States of America

**Peter Ruhdal Jensen**

Denmark

**Michael Jewett**

United States of America

**Zhengbing Jiang**

China

**Cheng Jin**

China

**Toru Jojima**

Japan

**Akinobu Kajikawa**

Japan

**Héla Kallel**

Tunisia

**Levente Karaffa**

Hungary

**Yasuhiko Kawakami**

United States of America

**Makoto Kawamukai**

Japan

**Yoshikazu Kawata**

Japan

**OLiver Kayser**

Germany

**Jay D. Keasling**

United States of America

**Isam Khalaila**

Israel

**Kieran Kilcawley**

Ireland

**Jin Kim**

United States of America

**Todd Klaenhammer**

United States of America

**Maria Klapa**

Greece

**Michiel Kleerebezem**

Netherlands

**Mario Klimacek**

Austria

**Mattheos Koffas**

United States of America

**Akihiko Kondo**

Japan

**Chandraraj Krishnan**

India

**Jens Olaf Krömer**

Australia

**Wolfgang Kroutil**

Austria

**Oscar Kuipers**

Netherlands

**Gotthard Kunze**

Germany

**Junichi Kurawaki**

Japan

**Christine Lang**

Germany

**Siegmund Lang**

Germany

**Philippe Langella**

France

**Alvaro R. Lara**

Mexico

**Christer Larsson**

Sweden

**Dong-Yup Lee**

Singapore

**David Lee**

United Kingdom

**Jung-Kul Lee**

Korea, South

**Won-Jae Lee**

Korea, South

**Peter Lee**

Japan

**Kim Jye Lee Chang**

Australia

**Livia Leoni**

Italy

**Yuezhong Li**

China

**Yin Li**

China

**Gunnar Liden**

Sweden

**Mark Liles**

United States of America

**Carol Sze Ki Lin**

Hong Kong

**Geoff Lin-Cereghino**

United States of America

**Dirk Linke**

Norway

**Hong Liu**

United States of America

**Yanhong Liu**

United States of America

**Agustin Lopez Munguia**

Mexico

**Nicole Lorenz**

Germany

**Julia Lorenzo**

Spain

**Joen Luirink**

Netherlands

**Tina Lütke-Eversloh**

Germany

**Robert Mach**

Austria

**Astrid Rosa Mach-Aigner**

Austria

**Søren Madsen**

Denmark

**Pavlos Makridis**

Greece

**Pin-Ching Maness**

United States of America

**Robert Mans**

Netherlands

**Esteban Marcellin**

Australia

**Eric Marechal**

France

**Antoine Margeot**

France

**Abelardo Margolles**

Spain

**Monica Marin**

Uruguay

**Ian Marison**

Ireland

**Viggo Marteinsson**

Iceland

**Jun-ichi Maruyama**

Japan

**Hans Marx**

Austria

**Antonio Marzocchella**

Italy

**Sergey Mashko**

Russian Federation

**Emilia Matallana**

Spain

**Sabine Matallana-Surget**

France

**Diethard Mattanovich**

Austria

**Michael Maurer**

Austria

**Ana Mazotto**

Brazil

**Olivia McAuliffe**

Ireland

**Rosli Md Illias**

Malaysia

**Wilfried Meijer**

Spain

**Ana Mendes-Ferreira**

Portugal

**Jeff Mertens**

United States of America

**Rolandas Meskys**

Lithuania

**Stanislav Miertus**

Slovakia

**Elizabeth Miller**

United States of America

**Norihiko Misawa**

Japan

**Wilfrid Mitchell**

United Kingdom

**Axel Mogk**

Germany

**Venkata Mohan**

India

**Ana Moldes**

Spain

**Francesco Molinari**

Italy

**Manuel Montero**

Spain

**Diego Mora**

Italy

**David Moreno**

Sweden

**Shigeru Morimura**

Japan

**Tomotoake Morita**

Japan

**John Morrissey**

Ireland

**Uffe Mortensen**

Denmark

**Matthias Mueller**

Germany

**Rolf Müller**

Germany

**Cormac Murphy**

Ireland

**Les Nagata**

Canada

**Kenneth Narva**

United States of America

**Antonino Natalello**

Italy

**Gennadi Ivanovich Naumov**

Russian Federation

**Rodrigo Navia**

Chile

**Peter Neubauer**

Germany

**Horst Neve**

Germany

**Bernd Nidetzky**

Austria

**Jens Nielsen**

Sweden

**Jens Nielsen**

Denmark

**Pablo Ivan Nikel**

Spain

**Sunee Nitisinprasert**

Thailand

**David Nix**

United States of America

**Stephan Noack**

Germany

**Norihisa Noguchi**

Japan

**Philippe Noirot**

France

**Michal Obuchowski**

Poland

**Rachel Ohana**

United States of America

**Kenji Okano**

Japan

**Marco Oldiges**

Germany

**Giuseppe Olivieri**

Netherlands

**Mehmet Orman**

United States of America

**Sahlan Ozturk**

Turkey

**Santosh Kumar Padhi**

India

**Tiina Pakula**

Finland

**Laura Palomares**

Mexico

**Amulya Panda**

India

**Potjamas Pansri**

Denmark

**Deepak Pant**

Belgium

**Maria Papagianni**

Greece

**Christos Papaneophytou**

Greece

**Wilson Parawira**

Zimbabwe

**Sunghoon Park**

Korea, South

**Ermenegilda Parrilli**

Italy

**Volkmar Passoth**

Sweden

**Mario Pedraza-Reyes**

Mexico

**Siwu Peng**

United States of America

**Spela Peternel**

Slovenia

**Maria Julia Pettinari**

Argentina

**Peter Philippsen**

Switzerland

**Konstantin Piatkov**

United States of America

**Harald Pichler**

Austria

**David Pollard**

United States of America

**Danilo Porro**

Italy

**Gyorgy Posfai**

Hungary

**Radha Prasanna**

India

**Anna Maria Puglia**

Italy

**Peter J. Punt**

Netherlands

**Qingsheng Qi**

China

**Lake-Ee Quek**

Australia

**Amparo Querol**

Spain

**Gabor Rakhely**

Hungary

**Octavio Ramirez**

Mexico

**Bruce Ramsay**

Canada

**Viviana A. Rapisarda**

Argentina

**R. George Ratcliffe**

United Kingdom

**Kenneth Reardon**

United States of America

**John Regan**

United States of America

**Alberto Reis**

Portugal

**Jose L. Revuelta**

Spain

**Ezio Ricca**

Italy

**Peter Richard**

Finland

**Michel Rigoulet**

France

**Ian Roberts**

United Kingdom

**Gilles Robitaille**

Canada

**Isabel Rocha**

Portugal

**Ligia Rodrigues**

Portugal

**Stefan Rokem**

Israel

**Avena Ross**

United States of America

**R. Paul Ross**

Ireland

**Maddalena Rossi**

Italy

**Warren Ruder**

United States of America

**Anne Ruffing**

United States of America

**Hector A. Ruiz**

Mexico

**Françoise Rul**

France

**Karl Rumbold**

South Africa

**Peter Ruoff**

Norway

**Laura Ruohonen**

Finland

**Luis Rodrigo Saa**

Ecuador

**Paolo Saccardo**

Spain

**Toshiyuki Sakaki**

Japan

**Tulasi Satyanarayana**

India

**Michael Sauer**

Austria

**Nicolas Sauvageot**

France

**Shigeki Sawayama**

Japan

**Anett Schallmey**

Germany

**Chiara Schiraldi**

Italy

**Monika Schmoll**

Austria

**Konstantin Schneider**

Germany

**Anja Schüffler**

Germany

**Helmut Schwab**

Austria

**Thomas Schweder**

Germany

**Gerd M. Seibold**

Germany

**Bernhard Seiboth**

Austria

**David Sela**

United States of America

**Joaquin Seras**

Spain

**Yanina Sevastsyanovich**

United Kingdom

**Lei Shao**

China

**Joseph Shiloach**

United States of America

**Kazuyuki Shimizu**

Japan

**Hiroshi Shimizu**

Japan

**Andriy Sibirny**

Ukraine

**Verena Siewers**

Sweden

**Filomena Silva**

Portugal

**Humberto de Jesus Silva**

Argentina

**Steven Singer**

United States of America

**Jean-Claude Sirard**

France

**Arne Skerra**

Germany

**Nadia Skorupa Parachin**

Brazil

**Anita Slavica**

Croatia

**Christian Solem**

Denmark

**Young-Chae Song**

Korea, South

**Yuan Song**

China

**Jens Laurids Sørensen**

Denmark

**Maria João Sousa**

Portugal

**Oliver Spadiut**

Austria

**Giuseppe Spano**

Italy

**Michael Spector**

United States of America

**Edzard Spillner**

Germany

**Georg Sprenger**

Germany

**Alok Srivastava**

India

**Matthias Steiger**

Austria

**Heux Stephanie**

France

**Ivana Strahinic**

Serbia

**Kumar Sudesh**

Malaysia

**Birgitta Sundström**

Sweden

**Sabine Suppmann**

Germany

**Izabela Swiecicka**

Poland

**Chakrit Tachaapaikoon**

Thailand

**Kaoru Takegawa**

Japan

**Yutaka Tamaru**

Japan

**Dan Tan**

China

**Huarong Tan**

China

**Chandresh Thakker**

India

**Johan Thevelein**

Belgium

**Anne Thierry**

France

**Nuttha Thongchul**

Thailand

**Oliver Thum**

Germany

**Elia Tomas-Pejo**

Sweden

**Hiep Tran**

United States of America

**Maria Luisa Tutino**

Italy

**Nicola A. Uccella**

Italy

**Ugutz Unzueta**

Spain

**Maria C. Urdaci**

France

**Francisco Valero**

Spain

**Daniel Vallera**

United States of America

**Peter van Baarlen**

Netherlands

**Laura van der Straat**

Netherlands

**Jan Maarten van Dijl**

Netherlands

**Jos van Putten**

Netherlands

**Douwe van Sinderen**

Ireland

**Klaas Vandenbroucke**

Belgium

**Navin Varadarajan**

United States of America

**Anikó Várnai**

Norway

**Vinod Vathipadiekal**

United States of America

**Esther Vázquez**

Spain

**Karthik Veeravalli**

United States of America

**Ciddi Veeresham**

India

**Salvador Ventura**

Spain

**Henrique Vicente**

Portugal

**Claudia Vickers**

Australia

**John Villadsen**

United States of America

**Marie-Joelle Viroelle**

France

**Michelle Visser**

United States of America

**Varaporn Vuddhakul**

Thailand

**Graeme Walker**

United Kingdom

**Fiona Walsh**

Ireland

**Xuedong Wang**

China

**Ren Wei**

Germany

**Paul Weimer**

United States of America

**Brian Weiss**

United States of America

**Jianping Wen**

China

**Volker F. Wendisch**

Germany

**Thomas West**

United States of America

**Ian Wheeldon**

United States of America

**Stephanie Willing**

United Kingdom

**Keehoon Won**

Korea, South

**Luet Wong**

United Kingdom

**Han Min Woo**

Korea, South

**Mhairi Workman**

Denmark

**Jin Chuan Wu**

Singapore

**Florian Wurm**

Switzerland

**Xudong Xu**

China

**Alexander Yakunin**

Canada

**Hongwei Yu**

China

**Vilem Zachleder**

Czech Republic

**Natalia Zakataeva**

Russian Federation

**Jurgen Zanghellini**

Austria

**Jürgen Zanghellini**

Austria

**Rintze Zelle**

United States of America

**Xueli Zhang**

China

**Feng Zhao**

China

**Shengde Zhou**

United States of America

**Wolfgang Zimmermann**

Germany

